# Joint-level responses to tofacitinib and methotrexate: a post hoc analysis of data from ORAL Start

**DOI:** 10.1186/s13075-023-03144-1

**Published:** 2023-09-29

**Authors:** Adrian Ciurea, Oliver Distler, Kenneth Kwok, Hyejin Jo, Lisy Wang, Tim Killeen, Caroline Ospelt, Mojca Frank Bertoncelj

**Affiliations:** 1 Department of Rheumatology, Center of Experimental Rheumatology, University Hospital Zürich, University of Zürich, Zürich, Switzerland; 2 Inflammation & Immunology, Pfizer Inc., New York, NY USA; 3 Inflammation & Immunology, Pfizer Inc., Groton, CT USA; 4 Inflammation & Immunology, Pfizer AG, Schärenmoosstrasse 99, 8052 Zürich, Switzerland; 5 BioMed X Institute, Heidelberg, Germany

**Keywords:** Tofacitinib, Rheumatoid arthritis, Methotrexate, Radiographic, Joint

## Abstract

**Background:**

Rheumatoid arthritis (RA) has a variable impact on different synovial joints, with inflammation being more commonly observed in some joints than others. Emerging evidence suggests that the anatomical variation in pathophysiology could result in differential responses to treatments across the joints, both within and between modes of action. This analysis aimed to characterize joint-specific responses to tofacitinib and methotrexate monotherapy in patients with RA.

**Methods:**

This was a post hoc analysis of data from the phase III trial ORAL Start (NCT01039688), in methotrexate-naïve patients with RA. A paired joint pathology score (PJPS), derived from bilateral tender/swollen joint counts, was calculated. The percentage change from baseline in PJPS (%∆PJPS) and treatment-specific responses (tofacitinib 5 and 10 mg twice daily [BID] vs methotrexate; tofacitinib 5 vs 10 mg BID) for each patient joint pair, except for those with baseline/post-baseline PJPS = 0, were calculated at month 3, month 6, and month 12. Radiographic progression was similarly assessed using the Modified Total Sharp Score at month 6 and month 12.

**Results:**

In methotrexate-naïve patients, differences in %∆PJPS demonstrated greater responses with tofacitinib vs methotrexate in most joint locations. Lesser responses with tofacitinib vs methotrexate were observed in most joints of the feet, particularly at month 12. Despite this, radiographic progression at month 12 was significantly worse in the foot (and metacarpophalangeal) joints of patients receiving methotrexate vs tofacitinib.

**Conclusion:**

We observed variation in joint-specific responses with tofacitinib and methotrexate monotherapy. Despite a proximal–distal efficacy gradient, with better clinical responses in the feet, patients receiving methotrexate monotherapy demonstrated more radiographic progression in the foot joints compared with those receiving tofacitinib. These findings suggest that body site- and therapy-specific characteristics may interact to produce differential treatment responses.

**Trial registration:**

ClinicalTrials.gov, NCT01039688.

**Supplementary Information:**

The online version contains supplementary material available at 10.1186/s13075-023-03144-1.

## Background

Rheumatoid arthritis (RA) is a chronic, systemic, autoimmune disease characterized by synovial inflammation and joint destruction [1]. In RA, the synovial tissue is heavily hyperplastic, with the expansion of fibroblast-like synoviocytes [2–4], insufficiency of tissue-resident macrophages [5, 6], and infiltration of diverse immune cells [2, 7], resulting in inflammation, joint swelling, and structural damage [8]. While the body distribution of joints involved in RA is classically symmetrical [9], synovial joints are affected variably, with inflammation more commonly observed in some joints (e.g., hand metacarpophalangeal [MCP] and wrist joints), compared with others (e.g., distal interphalangeal [DIP] joints) [10, 11].

Evidence is emerging to suggest that an epigenetic component may contribute to joint-specific changes in the pathogenesis of RA and, thus, may modify treatment response [9, 12]. For example, site-specific differences have been observed in the transcriptome, epigenome, and function of synovial fibroblasts from different joints [13]. This includes evidence that some joints in RA show differential epigenetic modifications of genes encoding biologic pathways, such as interleukin-6 signaling via the Janus kinase (JAK)-signal transducer and activator of transcription (STAT) pathway [9, 12].

Such genetic gradients may be imprinted during embryogenesis and manifest along body and limb axes, for instance, from proximal to distal along the whole body (e.g., shoulder, hip, knee, and foot) or down the upper limb (e.g., shoulder, elbow, wrist, hand MCP, and hand proximal interphalangeal [PIP] joints) [14]. If such locational variation in pathophysiology results in differential responsiveness to specific pathways or cytokine inhibitors, optimal RA therapy choices could be guided, in part, by joint involvement patterns. However, in clinical practice, therapeutic response is measured as changes in total disease burden rather than as changes in symptomatic synovitis in individual joints [15]. Accordingly, randomized controlled trials (RCTs) in RA generally report summated 68/66-joint counts or, alternatively, 28-joint counts based on the American College of Rheumatology (ACR) and Disease Activity Score in 28 joints (DAS28) criteria. Summation and the fact that the 28-joint counts omit ankle and foot joints [16] potentially mask anatomical variation in therapeutic response within and between modes of action [17–20].

Tofacitinib is an oral JAK inhibitor for the treatment of RA. The objective of this post hoc analysis was to characterize joint-specific responses to tofacitinib and methotrexate administered as monotherapy in the phase III ORAL Start RCT. This trial was chosen due to the availability of the 66/68-joint count and its head-to-head design and because it was conducted in methotrexate-naïve patients, allowing inferences to be drawn on the effect of drugs with different modes of action on potential site-specific target pathway involvement. The use of the ORAL Start dataset also allowed the evaluation of any joint-specific radiographic responses to methotrexate and JAK inhibition with tofacitinib.

## Methods

### Study design

ORAL Start (NCT01039688) was a 24-month, double-blind phase III RCT designed to evaluate the clinical, structural, and safety outcomes of tofacitinib vs methotrexate, both administered as monotherapy, in methotrexate-naïve patients. The full study design of ORAL Start has been published previously [21]. Briefly, patients were ≥ 18 years old with active RA according to the ACR 1987 revised criteria [22], with ≥ 6 tender or painful joints (out of 68 joints examined) and ≥ 6 swollen joints (out of 66 joints examined). Patients also had ≥ 3 distinct joint erosions detected on hand and wrist or foot radiographs. Patients were randomized 2:2:1 to receive tofacitinib 5 mg twice daily (BID), tofacitinib 10 mg BID, or methotrexate (starting dose 10 mg/week, with increments of 5 mg/week every 4 weeks to 20 mg/week by week 8). The coprimary endpoints assessed at month 6 were the proportion of patients achieving an ACR70 response (≥ 70% response on ACR criteria) and changes from baseline in the Modified Total Sharp Score (mTSS).

The study was conducted in accordance with the Declaration of Helsinki, International Council for Harmonisation Guidelines for Good Clinical Practice, and local country regulations. The institutional review board at each study center and relevant independent ethics committees approved the study, and all patients provided written informed consent.

### Post hoc analysis of joint-specific responses to tofacitinib and methotrexate monotherapy in ORAL Start

Data for tender/swollen joint counts (measured at screening, baseline, and each visit up to month 12) were assessed in the left and right synovial joints for a given anatomical location. Further information on the data collection and the specific joints assessed can be found in Additional file 1. Data from the left and right synovial joints were pooled, an approach justified by the assumption that the genetic environments of the left and right joints are identical. The resulting data are described as a paired joint pathology score (PJPS). The PJPS ranges from 0 (neither side swollen nor tender) to 4 (both sides swollen and tender) for each joint pair. Each of the component joint pairs making up the 68/66-joint count was included, resulting in PJPS data for 33 joint pairs; the hip was excluded from the analysis as joint swelling is not assessed.

The percentage change from baseline in PJPS (%∆PJPS) was assigned based on the combination of baseline and post-baseline scores at months 3, 6, and 12. Treatment-specific responses for each joint were assessed by calculating the differences in mean %∆PJPS for each tofacitinib dose (5 or 10 mg BID) vs methotrexate. Similarly, tofacitinib dose-related responses for each joint were assessed by calculating the differences in mean %∆PJPS for tofacitinib 5 vs 10 mg BID. The PJPS was further broken down into its constituent components (tenderness score [0–2] and swelling score [0–2]) to provide insights into the response of these two manifestations of joint inflammation.

Radiographic progression in specific joint groups was determined using the constituents of the mTSS, a combined index of erosion scores and joint space narrowing (JSN) scores. The mTSS was used to assess five joint groups: hand MCP, hand PIP, wrist, foot interphalangeal (IP), and foot metatarsophalangeal (MTP) joints. In a similar manner to the PJPS previously described, a radiographic PJPS (rPJPS) was calculated for erosion scores, JSN scores, and total mTSS, derived from bilateral mTSS component/total scores in each joint group. For erosion scores, JSN scores, and total mTSS, least squares mean change from baseline in rPJPS (∆rPJPS) at month 12 was calculated for each joint group.

### Statistical analyses

All %∆PJPS data were summarized descriptively and presented as bar graphs and homunculi.

For ∆rPJPS, statistical comparisons between tofacitinib 5 and 10 mg BID and methotrexate were performed using a mixed model for repeated measures, adjusted for age, geographic region, RA disease duration, disease activity (measured by DAS28, erythrocyte sedimentation rate [DAS28-4(ESR)]; only the subset of joints contributing to the DAS28 are used in the overall disease activity measures, for standardization purposes), and the baseline value of the endpoint. Similarly, subanalyses were performed for the erosion scores and JSN scores. Significance was defined as *P* < 0.05, and no adjustments for multiplicity were made.

Shift tables between baseline PJPS and post-baseline rPJPS for the foot and foot-MTP joints were produced using the quartile values (0, Q1, Q2, Q3, maximum) to assess whether baseline PJPS was predictive of post-baseline rPJPS.

## Results

### Patients

In ORAL Start, 958 patients were randomized, and 956 patients received treatment. Of these 956 patients, 373 received tofacitinib 5 mg BID, 397 received tofacitinib 10 mg BID, and 186 received methotrexate (mean dose 18.5 mg/week) [21].

Demographics and baseline characteristics of the main study population were generally similar across groups and have been reported previously [21].

**Fig. 1:**
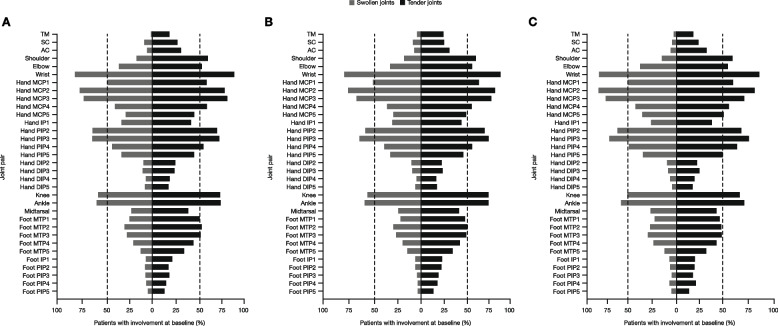
Proportion of patients with baseline joint pair involvement (PJPS > 0) in ORAL Start. Patients were receiving **A** tofacitinib 5 mg BID, **B** tofacitinib 10 mg BID, and **C** methotrexate; assessment of baseline joint pair involvement (PJPS > 0) included each of the component joint pairs making up the 68/66-joint count. The hip was excluded, as swelling was not assessed. The total number of patients assessed at baseline was *N* = 956 (tofacitinib 5 mg BID: *N* = 373; tofacitinib 10 mg BID: *N* = 397; methotrexate: *N* = 186). Refer to Table 1 for the number and proportion of patients with specific joint involvement at baseline. AC, acromioclavicular; BID, twice daily; DIP, distal interphalangeal; IP, interphalangeal; MCP, metacarpophalangeal; MTP, metatarsophalangeal; *N*, number of patients assessed at baseline; PIP, proximal interphalangeal; PJPS, paired joint pathology score; SC, sternoclavicular; TM, temporomandibular

In this post hoc analysis, data were available for 33 joint pairs. For each joint assessed, the percentage of patients with tender/swollen joints at baseline was generally similar for a given joint pair across treatment arms (Table 1). Approximately 50% of patients or more had joint involvement in the shoulder, elbow, wrist, hand MCP, most hand PIP joints, knee, ankle, and most medial foot MTP joints (Table 1). The frequency of tenderness and swelling in each joint pair across treatment groups was also assessed at baseline (Fig. 1). Generally, swelling was less frequent than tenderness, but followed a similar pattern. Tender/swollen joints were less frequent in the temporomandibular (TM), sternoclavicular (SC), and acromioclavicular (AC) joints, as well as in the hand DIP and foot IP joints (Table 1, Fig. 1).

**Table 1:** Baseline joint assessment in ORAL Start

	**Tofacitinib** **5 mg BID,** ***N* = 373**	**Tofacitinib** **10 mg BID,** ***N* = 397**	**Methotrexate, *N* = 186**
Total number of joints, mean^a^			
Tender joints	25.7	25.1	25.4
Swollen joints	16.3	15.6	16.8
mTSS and subcomponents, mean			
mTSS	19.1	17.9	16.1
Erosion score	9.1	9.1	8.4
JSN score	10.0	8.8	7.7
Patients with tender/swollen joints, *n* (%)			
TM	71 (19.0)	100 (25.2)	33 (17.7)
SC	104 (27.9)	103 (25.9)	43 (23.1)
AC	117 (31.4)	127 (32.0)	59 (31.7)
Shoulder	226 (60.6)	235 (59.2)	109 (58.6)
Elbow	208 (55.8)	228 (57.4)	106 (57.0)
Wrist	345 (92.5)	358 (90.2)	167 (89.8)
Hand MCP1	235 (63.0)	263 (66.2)	119 (64.0)
Hand MCP2	314 (84.2)	339 (85.4)	164 (88.2)
Hand MCP3	317 (85.0)	320 (80.6)	152 (81.7)
Hand MCP4	228 (61.1)	233 (58.7)	115 (61.8)
Hand MCP5	186 (49.9)	205 (51.6)	106 (57.0)
Hand IP1	171 (45.8)	184 (46.3)	75 (40.3)
Hand PIP2	282 (75.6)	290 (73.0)	139 (74.7)
Hand PIP3	280 (75.1)	312 (78.6)	152 (81.7)
Hand PIP4	219 (58.7)	235 (59.2)	126 (67.7)
Hand PIP5	181 (48.5)	193 (48.6)	97 (52.2)
Hand DIP2	96 (25.7)	94 (23.7)	41 (22.0)
Hand DIP3	97 (26.0)	99 (24.9)	46 (24.7)
Hand DIP4	74 (19.8)	71 (17.9)	37 (19.9)
Hand DIP5	70 (18.8)	72 (18.1)	33 (17.7)
Knee	282 (75.6)	303 (76.3)	127 (68.3)
Ankle	289 (77.5)	300 (75.6)	140 (75.3)
Midtarsal	154 (41.3)	177 (44.6)	86 (46.2)
Foot MTP1	198 (53.1)	194 (48.9)	89 (47.8)
Foot MTP2	206 (55.2)	205 (51.6)	90 (48.4)
Foot MTP3	201 (53.9)	198 (49.9)	92 (49.5)
Foot MTP4	172 (46.1)	172 (43.3)	84 (45.2)
Foot MTP5	132 (35.4)	140 (35.3)	61 (32.8)
Foot IP1	83 (22.3)	94 (23.7)	39 (21.0)
Foot PIP2	70 (18.8)	89 (22.4)	38 (20.4)
Foot PIP3	69 (18.5)	76 (19.1)	34 (18.3)
Foot PIP4	58 (15.5)	72 (18.1)	40 (21.5)
Foot PIP5	51 (13.7)	56 (14.1)	26 (14.0)

*AC* acromioclavicular, *BID* twice daily, *DIP* distal interphalangeal, *IP* interphalangeal, *JSN* joint space narrowing (score), *MCP* metacarpophalangeal, *MTP* metatarsophalangeal, *mTSS* Modified Total Sharp Score, *N* number of patients assessed at baseline, *n* number of patients with joint pair involvement (PJPS > 0) at baseline, *PIP* proximal interphalangeal, *PJPS* paired joint pathology score, *SC* sternoclavicular, *TM* temporomandibular

^a^Mean number of tender and swollen joints has been reported previously [21]

### Paired joint pathology scores

**Fig. 2:**
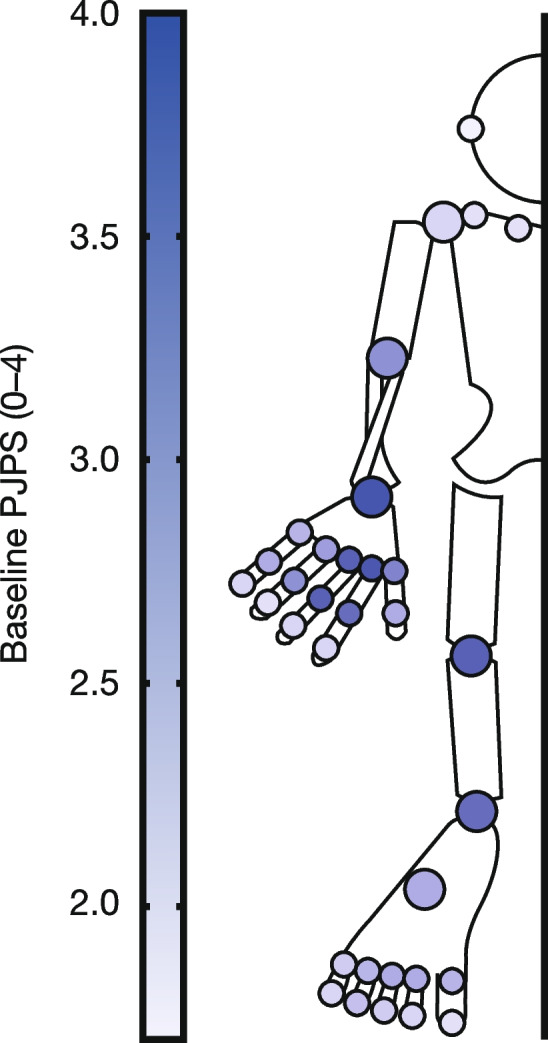
Mean baseline PJPS for selected joint pairs in ORAL Start (patients receiving tofacitinib 5 mg BID). Patients were receiving tofacitinib 5 mg BID; data represent the mean baseline PJPS in component joint pairs of the 68/66-joint count in patients with PJPS > 0 at baseline. The total number of patients assessed at baseline was *N* = 373. Refer to Table 1 for the number and proportion of patients with specific joint involvement at baseline. BID, twice daily; *N*, number of patients assessed; PJPS, paired joint pathology score

**Fig. 3:**
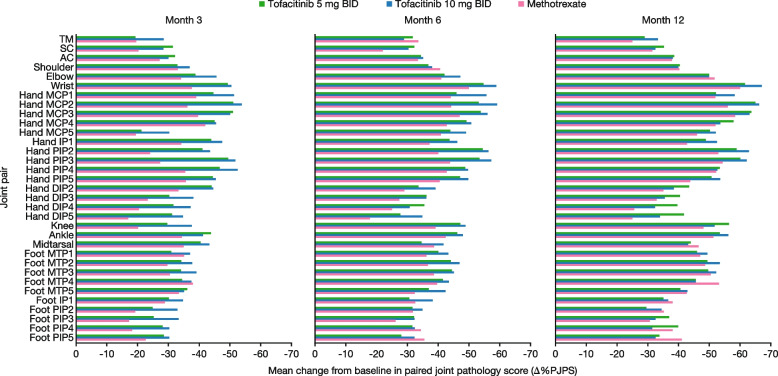
%∆PJPS in tender and swollen joints combined. Data represent mean %ΔPJPS at months 3, 6, and 12 in component joint pairs of the 68/66-joint count, in patients receiving tofacitinib 5 or 10 mg BID or methotrexate in ORAL Start. Number of patients assessed for each joint may vary. More negative %∆PJPS values represent greater efficacy in reducing signs of inflammation. %ΔPJPS, percentage change from baseline in PJPS; AC, acromioclavicular; BID, twice daily; DIP, distal interphalangeal; IP, interphalangeal; M, month; MCP, metacarpophalangeal; MTP, metatarsophalangeal; PIP, proximal interphalangeal; PJPS, paired joint pathology score; SC, sternoclavicular; TM, temporomandibular

Baseline PJPS was similar across the three treatment groups (Fig. 2 and Additional file 1: Fig. S1). Moreover, all joints showed a treatment response across treatment groups at month 3, as indicated by a negative %∆PJPS from baseline, with responses generally improving over time to month 12 (Fig. 3 and Additional file 1: Fig. S2). Greater %∆PJPS was generally seen in the joints with higher PJPS at baseline, which included the wrist, hand MCP, hand PIP, knee, ankle, and foot MTP joints (Fig. 3 and Additional file 1: Fig. S2).

### Therapy-specific differences in joint response

**Fig. 4:**
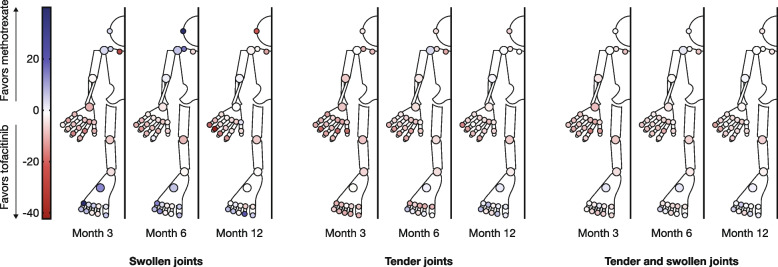
Mean difference in %∆PJPS between tofacitinib 5 mg BID and methotrexate in ORAL Start. Data demonstrate the mean differences in %∆PJPS for tofacitinib 5 mg BID at months 3, 6, and 12, minus the respective mean %∆PJPS for methotrexate in component joint pairs of the 68/66-joint count. %ΔPJPS, percentage change from baseline in PJPS; BID, twice daily; PJPS, paired joint pathology score

The mean differences in overall (i.e., tenderness and swelling combined) %∆PJPS at months 3, 6, and 12 showed better responses for both tofacitinib doses compared with methotrexate in the SC joint, most upper limb joints, knee, ankle, foot MTP2, and foot PIP3 joints (Fig. 4 and Additional file 1: Fig. S3). In the TM, AC, shoulder, and most foot joints, some consistent patterns were discernible (Fig. 4 and Additional file 1: Fig. S3). Overall %∆PJPS in the AC joint for tofacitinib 5 or 10 mg BID at month 3 was somewhat better than for methotrexate; an effect that waned to equivalence by month 12. At all three time points, both doses of tofacitinib demonstrated greater efficacy vs methotrexate in reducing tenderness in the AC joint, but there was a greater reduction in swelling at months 3 and 6 in the methotrexate group (Fig. 4 and Additional file 1: Fig. S3). A low proportion of patients had involvement of the TM joint (Table 1), and the results for swelling, tenderness, and combined %∆PJPS were mixed (Fig. 4 and Additional file 1: Fig. S3). In the shoulder, the effect of both tofacitinib doses and methotrexate were broadly similar, with a greater response to tofacitinib observed at some time points and for methotrexate at other time points for tender/swollen joints and overall %∆PJPS (Fig. 4 and Additional file 1: Fig. S3). The foot joints, from the midtarsal distally, showed a generally consistent pattern in terms of therapy-specific responses. Despite predominantly greater responses in overall %∆PJPS in the feet at month 3 for both tofacitinib doses, responses were greater with methotrexate in many foot joints by month 12 (Fig. 4 and Additional file 1: Fig. S3). In tender joints, the early benefit of both tofacitinib doses vs methotrexate was marked, but became less pronounced by month 6, particularly in the foot PIP joints for the tofacitinib 5 mg BID group. By month 12, methotrexate was generally equivalent or greater than tofacitinib 5 or 10 mg BID in many foot joints. In swollen foot joints, methotrexate induced consistently better responses vs tofacitinib 5 mg BID across all time points, while tofacitinib 10 mg BID generally showed a stronger response vs methotrexate at months 3 and 6, although by month 12 most swollen foot joints in the tofacitinib 10 mg BID group also showed a lesser response vs the methotrexate group (Fig. 4 and Additional file 1: Fig. S3).

Considering dose-dependency with tofacitinib, the mean differences in %∆PJPS showed that tofacitinib 10 mg BID generally conferred improved responses, in terms of joint swelling and tenderness combined, vs tofacitinib 5 mg BID for several joints, including the wrist, most hand MCP joints, hand IP, most hand PIP joints, ankle, and most foot MTP or IP joints, with differences between doses most pronounced in the first 3 months of treatment (data not shown).

### Radiographic progression at months 6 and 12

**Fig. 5:**
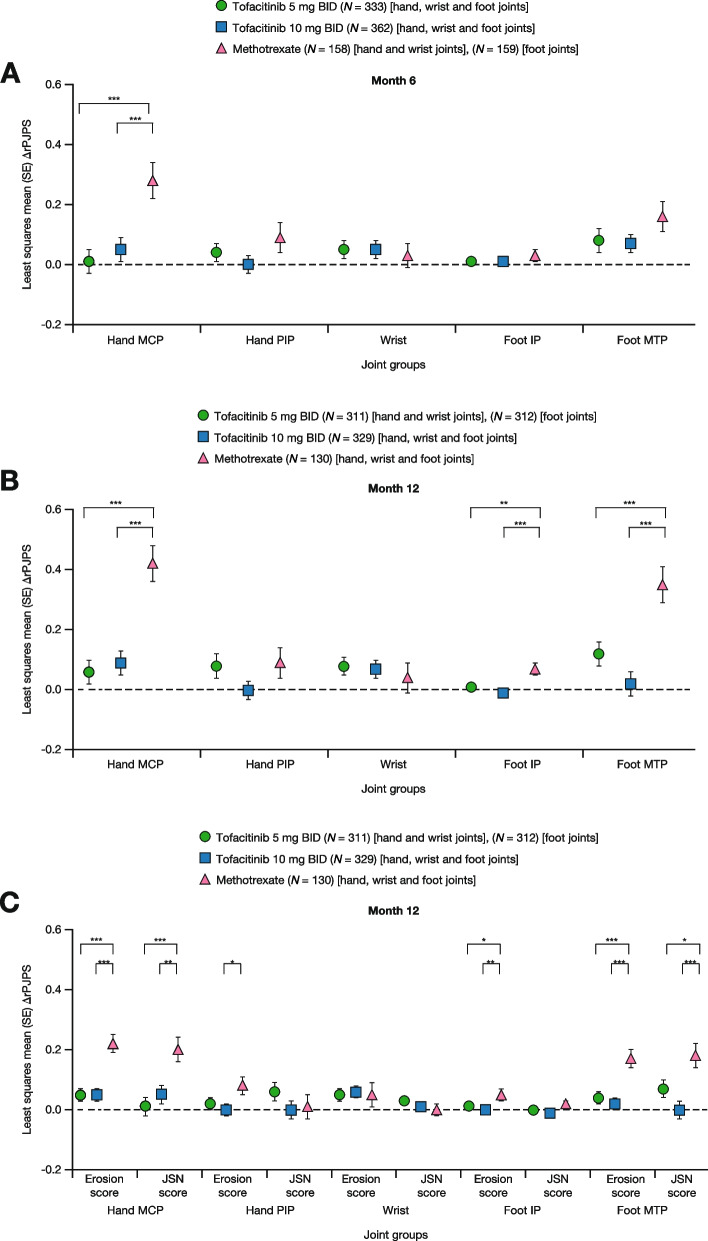
Least squares mean change from baseline in rPJPS in ORAL Start. Data represent total mTSS in five joint groups at **A** month 6 or **B** month 12 and **C** erosion and JSN scores at month 12, for patients receiving tofacitinib 5 or 10 mg BID or methotrexate. Data are based on a mixed model for repeated measures adjusted for age, geographic region, RA disease duration, disease activity (measured by DAS28-4(ESR)), and the baseline value of the endpoint. *P < 0.05; **P < 0.01; ***P < 0.001 tofacitinib 5 or 10 mg BID vs methotrexate. No multiplicity adjustments were made for statistical comparisons. ∆rPJPS, change from baseline in rPJPS; BID, twice daily; DAS28-4(ESR), Disease Activity Score in 28 joints, erythrocyte sedimentation rate; IP, interphalangeal; JSN, joint space narrowing (score); MCP, metacarpophalangeal; MTP, metatarsophalangeal; mTSS, Modified Total Sharp Score; *N*, number of patients assessed; PIP, proximal interphalangeal; RA, rheumatoid arthritis; rPJPS, radiographic paired joint pathology score; SE, standard error

Radiographic progression, as assessed by ∆rPJPS for erosion scores, JSN scores, and total mTSS, was minimal in all assessed joint groups (wrist, hand MCP, hand PIP, foot MTP, and foot IP joints) with either tofacitinib dose (Fig. 5A–C). The greater clinical %∆PJPS observed in some foot joints for methotrexate compared with either tofacitinib dose described above (Fig. 4) did not translate into improved radiographic outcomes in the foot at month 12. In fact, ∆rPJPS for total mTSS was significantly worse in the foot MTP and foot IP joints in those receiving methotrexate compared with either tofacitinib dose at month 12 (Fig. 5B). Similarly, ∆rPJPS in the hand MCP joints was significantly worse for methotrexate compared with either tofacitinib dose across erosion scores, JSN scores, and total mTSS at month 12 (Fig. 5B, C).

### Association between baseline paired joint pathology score and radiographic paired joint pathology score in foot joints

No significant associations were observed between baseline PJPS and rPJPS in the foot joints at month 12 in patients receiving tofacitinib 5 mg BID (*P* = 0.076), tofacitinib 10 mg BID (*P* = 0.773), or methotrexate (*P* = 0.867) (data not shown). Similar results were observed when only the MTP joints were considered (data not shown).

## Discussion

This post hoc analysis of data from ORAL Start investigated the effect of tofacitinib and methotrexate administered as monotherapy on joint-specific responses in methotrexate-naïve patients with active RA. While these findings come from a large, multicenter, clinical trial, our post hoc approach to analyzing joint-specific responses is new. This, and the considerable novelty of some findings, particularly those relating to clinical/radiographic decoupling in the feet of methotrexate-treated patients, mandates caution in interpretation and the need for confirmatory analyses in other datasets.

In this analysis, baseline joint involvement reflected the classical distribution of early RA as reported in real-world cohorts [23]. Patients generally had more tender than swollen joints, a relationship which appears to be associated with poorer response to therapy [24]. However, this should be interpreted in the context of the study design of ORAL Start, an RCT of tofacitinib in early RA that required patients to have ≥ 6 active joints for inclusion. In addition, swelling may be a more specific and less-sensitive marker of joint inflammation than subjective tenderness, with higher thresholds for clinical detection [25–27].

There was a wide variation in joint-specific responses to tofacitinib 5 or 10 mg BID and methotrexate up to month 12. Responses within all three treatment arms were generally strongest in the wrist, hand MCP, hand PIP, ankle, knee, and foot MTP joints, with generally lower responses observed elsewhere. This pattern of response is in keeping with greater anti-inflammatory responses in those joints with greater frequency of involvement at baseline. These joints presumably have greater inflammation and, consequently, the potential to respond to any potent anti-inflammatory therapy. In general, for overall %∆PJPS, tofacitinib 5 and 10 mg BID were more efficacious than methotrexate in most joint locations, except for some foot joints, at months 6 and 12, and the TM and shoulder joints for which greater responses to tofacitinib were observed at some time points and for methotrexate at others. While it is difficult to interpret the findings in the TM joint and shoulder since they represent single data points in isolated anatomical regions, the foot joint data appear to be consistent and robust.

We also report rapid, dose-dependent reduction in foot joint tenderness with tofacitinib vs methotrexate at month 3. However, this effect was not apparent for foot joint swelling, with a generally greater response following methotrexate vs tofacitinib 5 mg BID. In addition, although joint tenderness continued to improve with tofacitinib for most joints up to month 12 across all treatment groups, this was not maintained in the foot joints. Previous analyses of data from phase III RCTs of tofacitinib and baricitinib may offer insight into this finding. In ORAL Solo, tofacitinib monotherapy demonstrated rapid (as early as 2 weeks) and significant reductions in pain vs placebo in patients with RA and inadequate response to conventional synthetic or biologic disease-modifying antirheumatic drugs [28], while baricitinib with methotrexate demonstrated greater and more rapid pain relief than adalimumab with methotrexate in the phase III RCT RA-BEAM [29]. This superiority has been attributed in part to a putative JAK-STAT-mediated direct anti-nociceptive response, independent of anti-inflammatory effects [30, 31], which may account for the apparent dissociation observed here.

Our results also support the findings of previous studies that report the decoupling of clinical disease control from radiographic progression [32, 33]. Indeed, the greater clinical %∆PJPS response in the foot joints with methotrexate vs tofacitinib did not translate into improved radiographic outcomes at month 12, with significantly more radiographic progression observed in the hand MCP, foot MTP, and foot IP joints in patients receiving methotrexate vs tofacitinib 5 or 10 mg BID. Tofacitinib appears to confer good protection from radiographic progression despite a relatively weaker therapeutic effect on swelling in the joints of the feet. Taken together, the tentative data presented here add to an emerging consensus that radiographic progression at 6 months in patients otherwise responding well to methotrexate should prompt consideration of advanced therapies [32].

When assessing whether baseline joint involvement could be used as a predictor of later radiographic progression, we found that baseline clinical involvement (PJPS > 0) of the whole foot or MTP joints was not associated with increased rPJPS in these joint groups at month 12 in patients receiving either tofacitinib dose or methotrexate. These data suggest that the presence or absence of clinical baseline foot involvement cannot be exclusively relied upon as a predictor of radiographic progression in the feet. Additionally, the current findings suggest that particular attention should be paid to radiographic progression in the feet, as this may be distinct from the improvement of other clinical symptoms, as described above. In patients with early RA and significant foot involvement, further investigation is necessary to determine whether tofacitinib therapy in combination with methotrexate may be preferable to methotrexate monotherapy to minimize pain, swelling, and joint damage.

Previous studies have demonstrated that patients with high body mass index (BMI) may have lower radiographic progression, compared with low BMI [34–36], and therefore, high BMI may represent a less aggressive phenotype [35]. However, in ORAL Start, baseline BMI was very similar across patients receiving tofacitinib 5 mg BID, tofacitinib 10 mg BID, and methotrexate (mean 26.5–26.8 kg/m^2^ [Pfizer Inc., data on file]). Therefore, differences in BMI do not provide a sufficient explanation for the differing clinical and radiographic responses in patients receiving tofacitinib vs methotrexate.

Fibroblast-like synoviocytes from the hip and knee have previously been shown to have discrete transcriptomes, epigenetic markers, and JAK-STAT activation patterns, which may mediate the differential clinical responses observed within individual joints in patients with RA receiving tofacitinib [12]. Although the original study did not analyze fibroblast-like synoviocytes from the foot joints, consistent with the data presented here, it is plausible that JAK-STAT signaling differences within the MTP, IP, and PIP joints may contribute to altered therapeutic sensitivity to tofacitinib. Similarly, in some joints, notably the AC, SC, TM, knee, and some hand DIP joints, the %∆PJPS response to tofacitinib did not appear to be dose-dependent. While this may represent a ceiling effect in the case of the small DIP joints, which responded rapidly to tofacitinib, the relatively poor response of the foot joints may indicate lower sensitivity to JAK inhibition. Interestingly, while specific patterns of tender and swollen joint involvement are also observed in patients with psoriatic arthritis, manifestation of joint-specific effects does not appear to impact sensitivity to TNF inhibitor treatment [37]. This suggests that the reported joint-specific sensitivities of tofacitinib treatment may be due to modulation of the JAK-STAT pathway rather than the pathophysiologic cascades that promote inflammatory arthritis.

Several limitations must be considered in addition to the exploratory post hoc nature of the analysis. The key metrics (PJPS and rPJPS) were limited by the dichotomous nature and imperfect reliability of clinical joint examinations [38]. In addition, as %∆PJPS calculations excluded patients with no baseline and no post-baseline involvement, relatively few patients were included for less typical RA joints, such as the TM, SC, AC, hand DIP, and feet joints, and these analyses are, therefore, purely descriptive in nature. Some joints, due to anatomy, function, and individual differences, can harbor more inflammation before reaching the clinical threshold for tenderness and/or swelling.

While the 28-joint count correlates well with the 68/66-joint count [39], exclusive use of DAS28 to dictate disease activity state may conceal significant joint involvement of the lower limbs. Performing the full 66/68-joint count should be considered in clinical trials and registries to characterize whether a treatment demonstrates an anatomical response gradient and improve understanding of joint-level responses to drugs with various modes of action. Although monotherapy treatment arms are more useful than combination therapy for elucidation of the mode of action-specific response patterns, the homunculi presented herein do not represent “fingerprints” of tofacitinib or methotrexate, as even patients receiving monotherapy were likely to have been previously exposed to diverse therapies. Finally, the recommended dosage for RA is tofacitinib 5 mg BID or extended-release 11 mg once daily [40, 41]. Although tofacitinib 10 mg BID is not the approved treatment dosage for patients with RA in most countries, we believe that improving our understanding of joint-level responses with both tofacitinib doses is of clinical and scientific value and that data from patients who received the tofacitinib 10 mg BID dose should not be excluded from scientific investigation. Despite these limitations, this analysis is a rare example of “bench-to-post-hoc-to-bedside”, in which in vitro findings suggest a hypothesis that could be tested in existing clinical trial data.

## Conclusions

Several themes emerged from this post hoc analysis of joint-specific responses to tofacitinib and methotrexate in patients with early RA participating in ORAL Start. While tofacitinib 5 and 10 mg BID generally demonstrated greater response rates for tender and swollen joint pairs vs methotrexate, improved response rates in most foot joints were seen with methotrexate, particularly at later time points. Across several joint pairs assessed, radiographic progression was significantly worse with methotrexate vs tofacitinib. These findings suggest body site- and therapy-specific characteristics may interact to produce differential treatment responses; therefore, treatment response in different joints should continue to be monitored both clinically and radiographically. Clinical trial or registry datasets with other agents or in other arthritides (e.g., psoriatic arthritis) should be analyzed with similar joint-specific approaches to confirm or refute the findings observed here and provide further insights. Future research may also focus on single-cell “omics” studies of synovial cells and synovial tissues derived from the foot joints exposed to JAK inhibitors and/or methotrexate.

### Supplementary Information

**Additional file 1: **Supplementary information. **Fig. S1.** Mean baseline PJPS for selected joint pairs in ORAL Start in patients receiving **A** tofacitinib 10 mg BID and **B** methotrexate. **Fig. S2.** %ΔPJPS in **A** swollen joints and **B** tender joints. **Fig. S3.** Homunculus figures of the mean difference in %ΔPJPS between tofacitinib 10 mg BID and methotrexate in ORAL Start.

## Data Availability

Upon request, and subject to review, Pfizer will provide the data that support the findings of this study. Subject to certain criteria, conditions, and exceptions, Pfizer may also provide access to the related individual de-identified participant data. See https://www.pfizer.com/science/clinical-trials/trial-data-and-results for more information.

## Abbreviations

%∆PJPS: Percentage change from baseline in PJPS
AC: Acromioclavicular
ACR: American College of Rheumatology
ACR70 response: ≥ 70% Response on ACR criteria
BID: Twice daily
BMI: Body mass index
DAS28: Disease Activity Score in 28 joints
DAS28-4(ESR): DAS28, erythrocyte sedimentation rate
DIP: Distal interphalangeal
IP: Interphalangeal
JAK: Janus kinase
JSN: Joint space narrowing
MCP: Metacarpophalangeal
MTP: Metatarsophalangeal
mTSS: Modified Total Sharp Score
PIP: Proximal interphalangeal
PJPS: Paired joint pathology score
RA: Rheumatoid arthritis
RCT: Randomized controlled trial
rPJPS: Radiographic PJPS
SC: Sternoclavicular
SD: Standard deviation
SE: Standard error
STAT: Signal transducer and activator of transcription
TM: Temporomandibular
TNF: Tumor necrosis factor
