# Supplementary material for: Joint-level responses to tofacitinib and methotrexate: a post hoc analysis of data from ORAL Start
Source: Arthritis Res Ther. 2023 Sep 29;25:185. doi: 10.1186/s13075-023-03144-1 (PMC10540368; doi:10.1186/s13075-023-03144-1)
Supplement: Supplementary file 1 — Additional file 1: Supplementary information. Fig. S1. Mean baseline PJPS for selected joint pairs in ORAL Start in patients receiving A tofacitinib 10 mg BID and B methotrexate. Fig. S2. %ΔPJPS in A swollen joints and B tender joints. Fig. S3. Homunculus figures of the mean difference in %ΔPJPS between tofacitinib 10 mg BID and methotrexate in ORAL Start. [file 13075_2023_3144_MOESM1_ESM.pdf]

Supplementary information

Methodology

Tender/painful joint count (68)

Sixty-eight joints were assessed by a blinded joint assessor to determine the number of joints that were considered tender or painful. The response to pressure/motion on each joint was assessed using the following scale: Present/Absent/Not Done/Not Applicable (used for missing joints). Artificial joints were not assessed.

Swollen joint count (66)

The blinded joint assessor assessed joints for swelling using the following scale: Present/Absent/Not Done/Not Applicable (used for missing joints). Sixty-six joints were assessed for swelling. Artificial joints were not assessed.

Joints assessed

- Upper body: temporomandibular, sternoclavicular, and acromioclavicular.
- Upper extremity: shoulder, elbow, wrist (includes radiocarpal, carpal, and carpometacarpal considered as one unit), metacarpophalangeals (MCP I, II, III, IV, V), thumb interphalangeal (IP), proximal interphalangeals (PIP II, III, IV, V), and distal interphalangeals (DIP II, III, IV, V).
- Lower extremity: hip (left and right hip joints were not included in the swollen joint count), knee, ankle, tarsus (includes subtalar, transverse tarsal, and tarsometatarsal considered as one unit), metatarsophalangeals (MTP I, II, III, IV, V), great toe interphalangeal (IP), and proximal and distal interphalangeals combined (PIP II, III, IV, V).

Fig. S1 Mean baseline PJPS for selected joint pairs in ORAL Start in patients receiving **A** tofacitinib 10 mg BID and **B** methotrexate

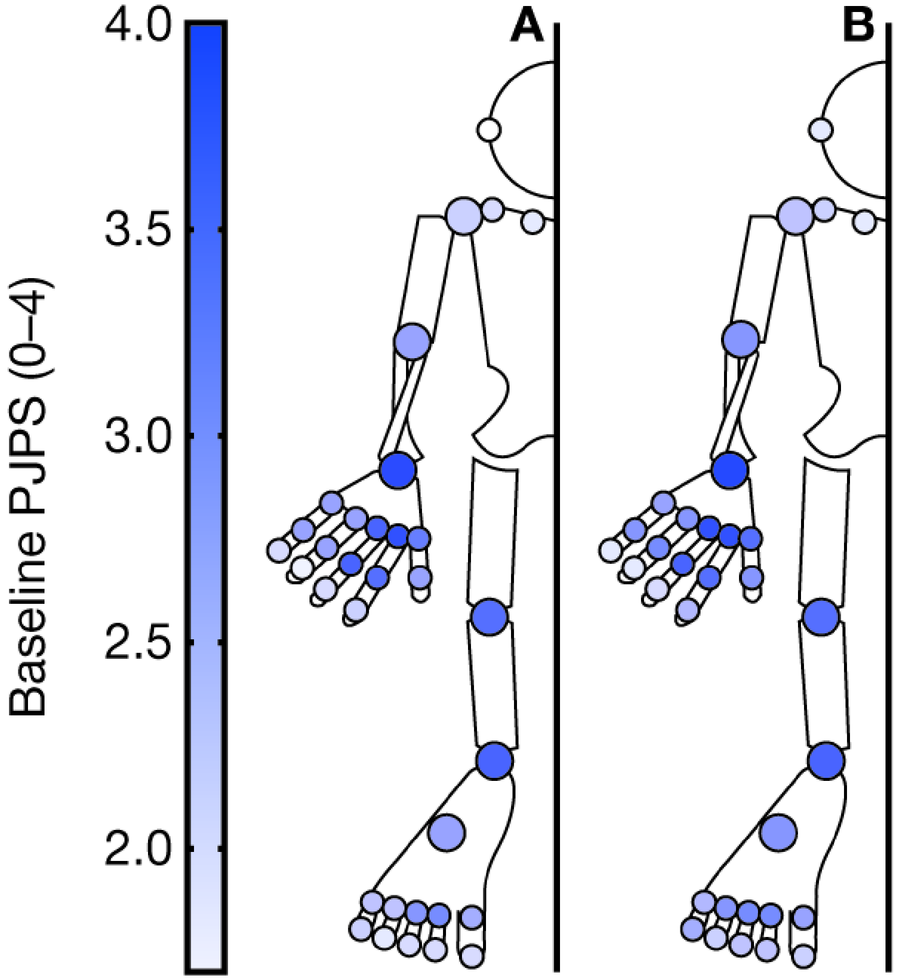

Data represent mean baseline PJPS in component joint pairs of the 68/66-joint count in patients with PJPS > 0 at baseline. Total number of patients assessed at baseline was N = 397 (tofacitinib 10 mg BID) and N = 186 (methotrexate). Refer to Table 1 for the number and proportion of patients with specific joint involvement at baseline. BID, twice daily; N, number of patients assessed; PJPS, paired joint pathology score

Fig. S2 %ΔPJPS in **A** swollen joints and **B** tender joints

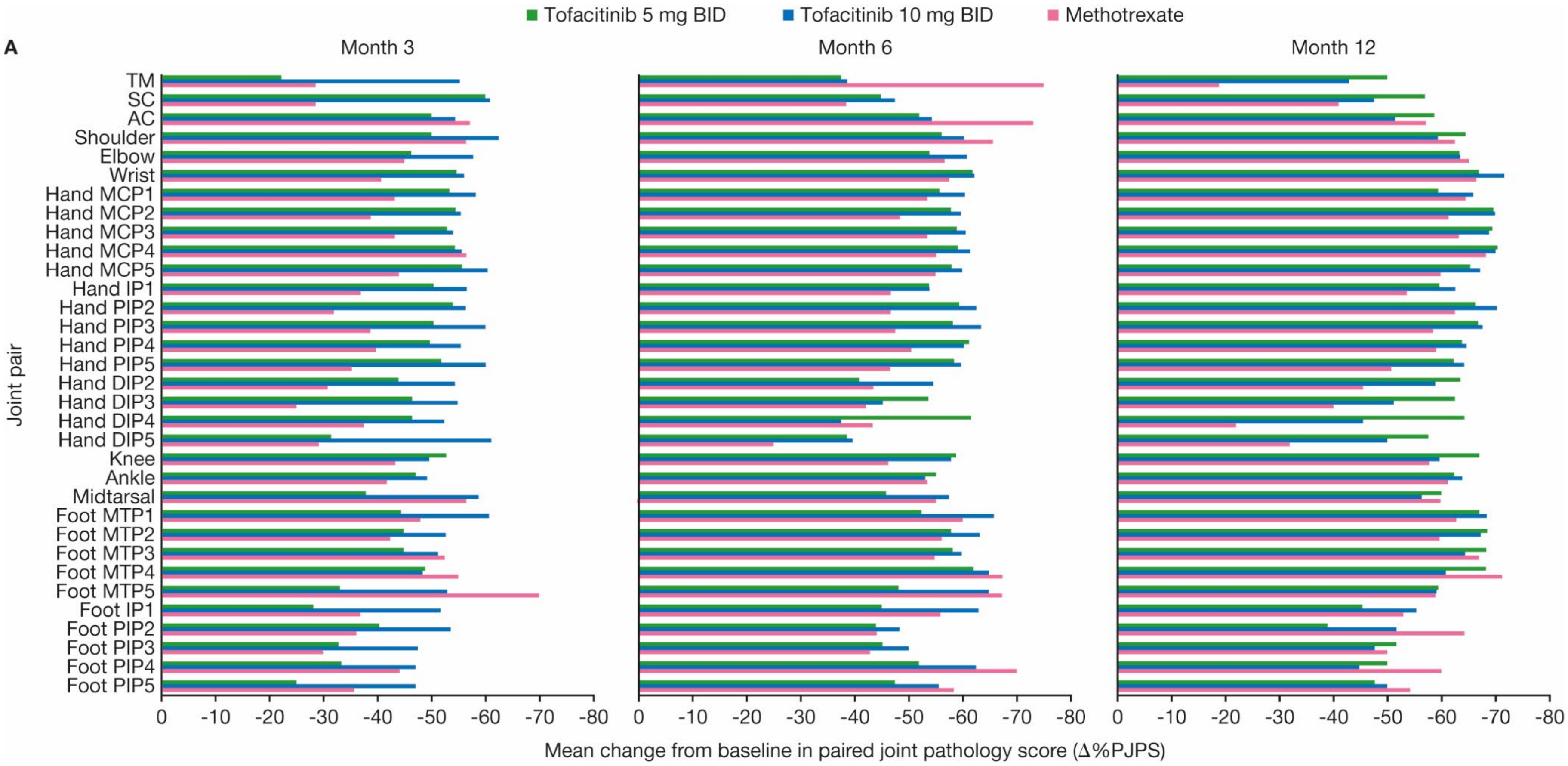

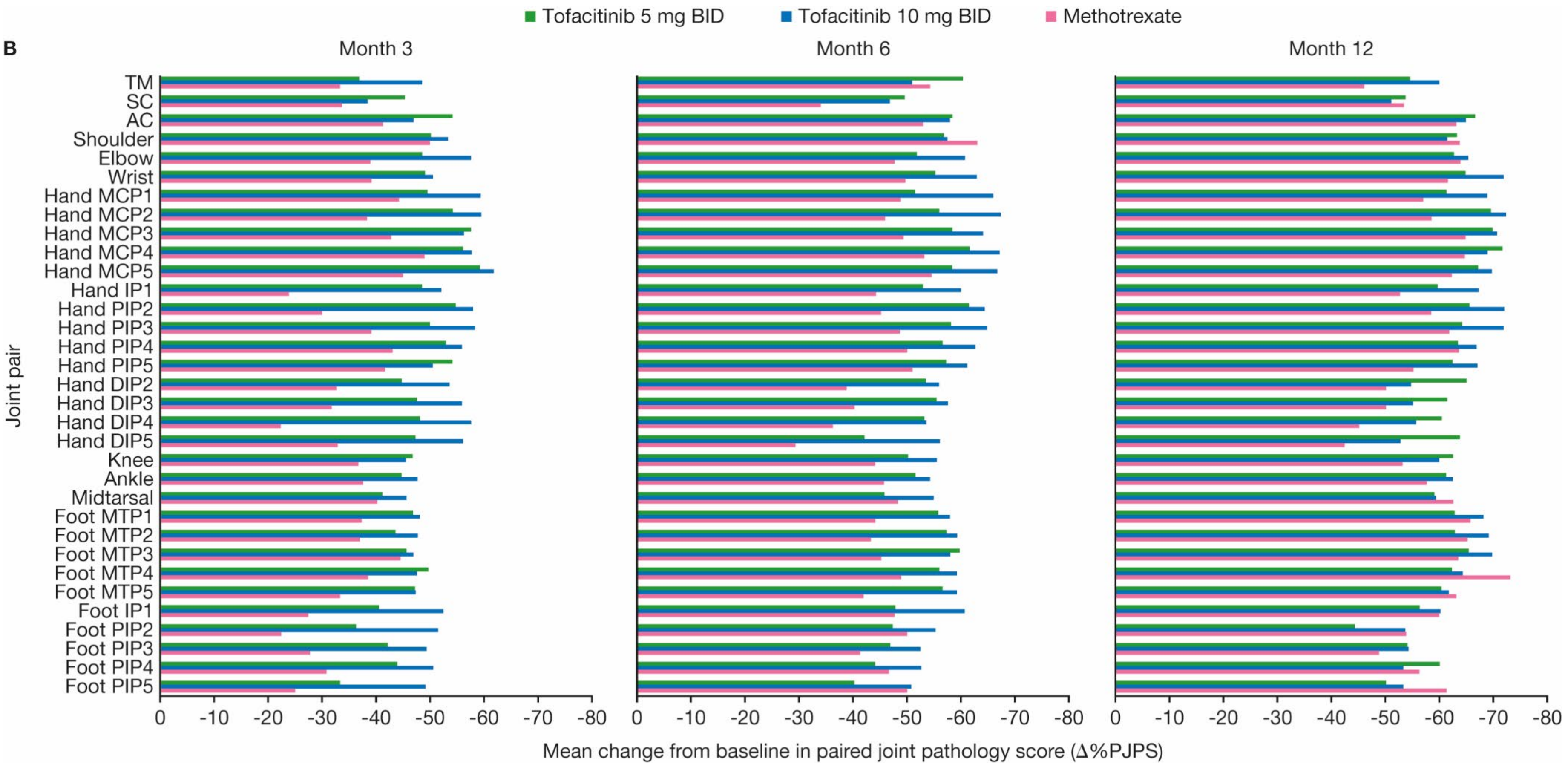

Data represent mean %ΔPJPS at months 3, 6, and 12 in component joint pairs of the 68/66-joint count in patients receiving tofacitinib 5 or 10 mg BID or methotrexate in ORAL Start. Number of patients assessed for each joint may vary. More negative %ΔPJPS values represent greater efficacy in reducing signs of inflammation. %ΔPJPS, percentage change from baseline in PJPS; AC, acromioclavicular; BID, twice daily; DIP, distal interphalangeal; IP, interphalangeal; MCP, metacarpophalangeal; MTP, metatarsophalangeal; PIP, proximal interphalangeal; PJPS, paired joint pathology score; SC, sternoclavicular; TM, temporomandibular

Fig. S3 Homunculus figures of the mean difference in %ΔPJPS between tofacitinib 10 mg BID and methotrexate in ORAL Start

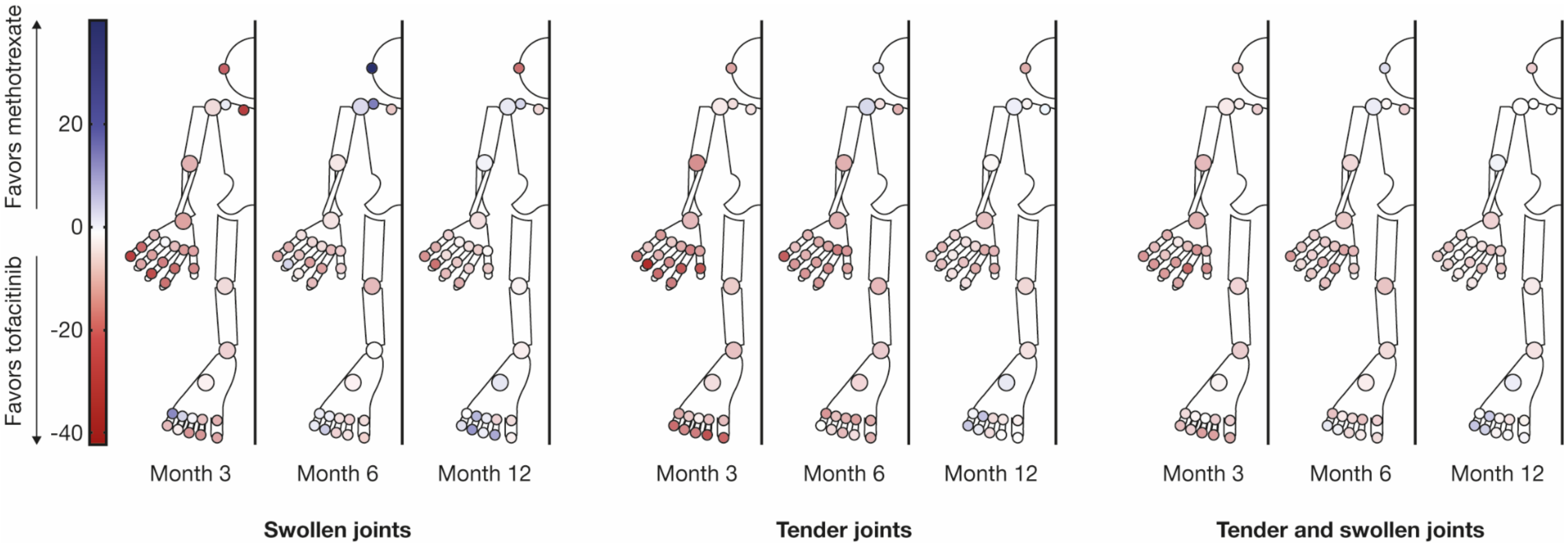

Data demonstrate mean differences in %ΔPJPS for tofacitinib 10 mg BID at months 3, 6, and 12, minus the respective mean %ΔPJPS for methotrexate in component joint pairs of the 68/66-joint count. %ΔPJPS, percentage change from baseline in PJPS; BID, twice daily; PJPS, paired joint pathology score
